# A serum metabolomics analysis reveals a panel of screening metabolic biomarkers for esophageal squamous cell carcinoma

**DOI:** 10.1002/ctm2.419

**Published:** 2021-05-06

**Authors:** Jiali Lv, Jialin Wang, Xiaotao Shen, Jia Liu, Deli Zhao, Mengke Wei, Xia Li, Bingbing Fan, Yawen Sun, Fuzhong Xue, Zheng‐Jiang Zhu, Tao Zhang

**Affiliations:** ^1^ Department of Biostatistics, School of Public Health Cheeloo College of Medicine, Shandong University Jinan China; ^2^ Institute for Medical Dataology Cheeloo College of Medicine, Shandong University Jinan China; ^3^ The Shandong Cancer Hospital Affiliated to Shandong University Jinan China; ^4^ Interdisciplinary Research Center on Biology and Chemistry, Shanghai Institute of Organic Chemistry Chinese Academy of Sciences Shanghai China; ^5^ Yanjing Medical College Capital Medical University Beijing China; ^6^ Tumor Preventative and Therapeutic Base of Shandong Province Feicheng People's Hospital Feicheng China

Dear Editor,

Endoscopy with iodine staining was widely used for esophageal cancer (EC) screening in high‐incidence area.^1^, ^2^ Most endoscopy screening‐positive population was found to develop esophageal epithelium lesion, and therefore endured higher risk for developing esophageal squamous cell carcinoma (ESCC) than normal population.^3^, ^4^ However, endoscopic screening may be too costly and invasive for large‐scale population, and non‐invasive biomarkers may be more applicable and cost effective for population‐based screening.^5^, ^6^

In this population‐based screening study, we aim to identify potential metabolic biomarkers for early screening of ESCC, and establish the optimal early ESCC screening model. Ultra‐performance liquid chromatography‐quadrupole time‐of‐flight mass spectrometry (UPLC‐QTOF/MS) was used to explore ESCC screening related metabolic biomarkers and profile difference between ESCC screening‐positive subjects and normal population. Detailed information on metabolites measurement and methods were provided in the Supporting Information.

In total, 1104 participants were included in this study (Table 1). Among the training dataset, ESCC screening‐positive subjects were more likely to be older males, with higher systolic blood pressure, higher proportion of smokers, alcohol drinker than healthy controls. No significant differences were found regarding sex, BMI, diastolic blood pressure, alcohol drinking between two groups.

**TABLE 1:** The baseline characteristics of training and validation dataset

Variables	Training (n = 662)			Validation (*n* = 442)		
	HC (*n* = 311)	PRCS (*n* = 351)	*p*‐Value	HC (*n* = 272)	PRCS (*n* = 170)	*p*‐Value
Age (year)	53.0 (7.6)	58.9 (7.2)	<0.001	53.3 (9.4)	58.1 (11.5)	<0.001
Females, *n* (%)	179 (57.6)	176 (50.1)	0.067	169 (62.1)	99 (45.0)	<0.001
BMI (kg/m^2^)	24.8 (3.4)	24.3 (7.6)	0.292	24.5 (3.4)	23.7 (3.1)	0.007
SBP (mm Hg)	129.8 (24.0)	134.4 (21.5)	0.009	130.8 (18.6)	133.8 (22.9)	0.133
DBP (mm Hg)	84.2 (13.8)	84.3 (11.9)	0.900	83.0 (10.4)	84.2 (11.7)	0.262
Smoker, *n* (%) ^†^	54 (17.4)	94 (26.8)	0.005	34 (12.5)	45 (26.5)	<0.001
Drinker, *n* (%) ^†^	76 (24.4)	110 (31.3)	0.059	6 (2.2)	1 (0.6)	0.350
**Pathology**						
Esophagitis, *n* (%)	–	78 (22.2)	–	–	56 (32.9)	–
Mild dysplasia, *n* (%)	–	189 (53.8)	–	–	68 (40.0)	–
Moderate dysplasia, *n* (%)	–	41 (11.7)	–	–	33 (23.6)	–
Severe dysplasia, *n* (%)	–	15 (4.3)	–	–	5 (2.9)	–
TIS, *n* (%)	–	12 (3.4)	–	–	4 (2.4)	–
Invasive tumor, *n* (%)	–	16 (4.6)	–	–	4 (2.4)	–

Data are means ± SD, or *n* (%).

Abbreviations: BMI, body mass index; DBP, diastolic blood pressure; ESCC screening‐positive subjects, PRCS; HC, healthy control; SBP, systolic blood pressure; TIS, tumor in situ.

^†^Rate was calculated after removing missing value.

Principal component analysis shows a clear tendency of separation between two groups (Figure 1B). The tight clustering trend of QC samples indicates a good analytical reproducibility of this metabolomics study. Partial least‐squares discriminant analysis was used to investigate the metabolic profile difference between ESCC screening‐positive subjects and healthy controls. Figure 1C also demonstrates a clear separation between two groups. The *Q*2 regression line and all permutated *R*2 values show that this model had no risk of overfitting (Figure 1D).

**FIGURE 1:**
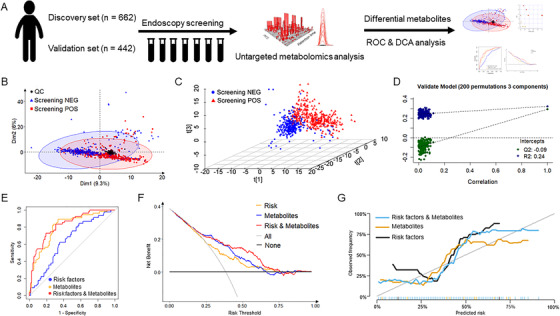
Metabolic profile analysis and early ESCC screening model. (A) Study design; (B) PCA score plot discriminating ESCC screening‐positive subjects and healthy controls; (C) PLS‐DA three‐dimensional scores plot discriminating ESCC screening‐positive subjects and healthy controls; (D) validation plot obtained from 200 permutation tests; (E) ROC curve for random forest model combing 14 metabolites; (F) decision curves for 14 metabolites to predict ESCC screening‐positive subjects; and (G) calibration curves for 14 metabolites to predict ESCC screening‐positive subjects

A total of 70 metabolites, with false discovery rate (FDR) smaller than 0.05 and the variable importance in projection (VIP) larger than 1, were selected as differential metabolites to predict ESCC screening‐positive subjects (Figure 2B and C). Among these differential metabolites, 14 differential metabolites were identified using reference standards, and the other 56 differential metabolites were interpreted according to their MS/MS spectra. Table S1 presents detailed information of 14 metabolites identified using reference standards. Due to the different level of metabolite identification, we built random forest models (RF models) with two combination of potential biomarkers, respectively.

**FIGURE 2:**
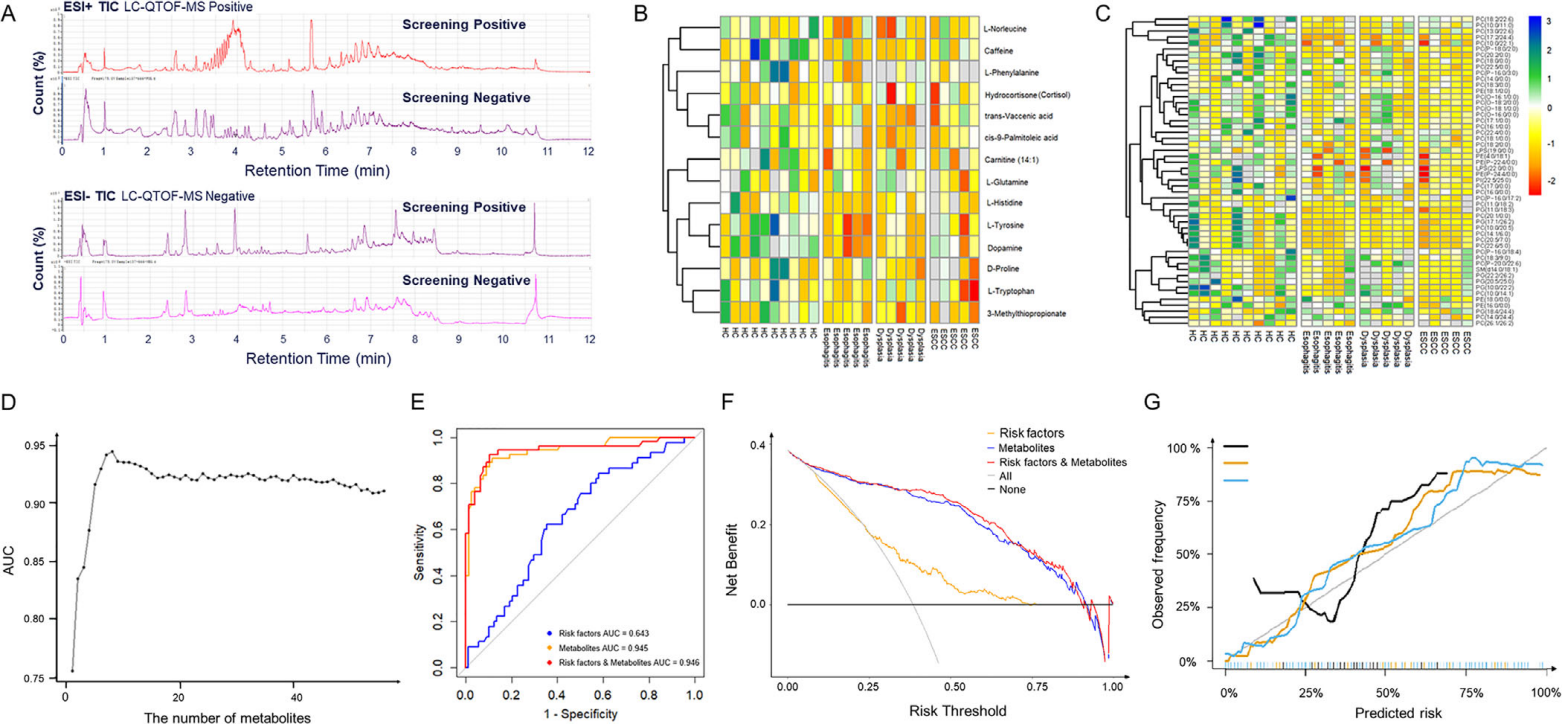
ESCC screening model composed with 8 metabolites. (A) The typical UPLC‐QTOF/MS chromatograms; (B) heatmap plot of 14 metabolites confirmed using standard references in the validation data; (C) heatmap plot of 56 metabolites interpreted according to their MS/MS spectra in the validation data; (D) best subset selection for metabolites interpreted according to their MS/MS spectra; (E) ROC analysis of random forest model for eight metabolites; (F) decision curves for eight metabolites to predict ESCC screening‐positive subjects; and (G) calibration curves for eight metabolites to predict ESCC screening‐positive subjects

Table 2 and Table S2 summarize RF models composed of 14 potential metabolic biomarkers to discriminate ESCC screening‐positive subjects in the validation dataset. As shown in Figure 1E, the RF model composed of traditional risk factors (age, sex, BMI, SBP, smoking, and alcohol drinking) demonstrates a poor performance in ESCC screening (AUC = 0.643, 95% CI 0.541–0.734). The combination of these 14 potential biomarkers had area under curve (AUC) value of 0.806 (95% CI: 0.728–0.878), with sensitivity of 87.3% (95% CI: 74.5–96.4%), specificity of 70.5% (95% CI: 59.0–82.1%), PPV of 67.6% (95% CI: 60.2–77.4%), and NPV of 88.7% (95% CI: 80.3–96.2). The RF model composed of 14 potential biomarkers shows good screening performance in different stages, especially in tumor in situ (TIS) and invasive cancer (AUC = 0.939, 95% CI 0.841–1.000).

**TABLE 2:** Random forest model composed 14 metabolic biomarkers to predict ESCC screening‐positive subjects

Model	N	AUC	Sensitivity	Specificity	PPV	NPV
Risk factors^†^		0.643 (0.541, 0.734)	0.756 (0.533, 0.933)	0.557 (0.341, 0.761)	0.466 (0.396, 0.575)	0.817 (0.738, 0.931)
Metabolites		0.806 (0.728, 0.878)	0.873 (0.745, 0.964)	0.705 (0.590, 0.821)	0.676 (0.602, 0.774)	0.887 (0.803, 0.962)
Metabolites and risk factors^†^		0.828 (0.755, 0.893)	0.782 (0.582, 0.927)	0.782 (0.615, 0.936)	0.719 (0.607, 0.878)	0.838 (0.753, 0.931)
Metabolites (by stages)						
Esophagitis	56	0.711 (0.596, 0.819)	0.800 (0.550, 1.000)	0.671 (0.316, 0.835)	0.365 (0.266, 0.519)	0.927 (0.867, 1.000)
Dysplasia	106	0.771 (0.665, 0.863)	0.839 (0.677, 0.968)	0.723 (0.554, 0.831)	0.532 (0.429, 0.651)	0.922 (0.859, 0.981)
TIS and Invasive cancer	8	0.939 (0.841, 1.000)	1.000 (1.000, 1.000)	0.902 (0.829, 1.000)	0.200 (0.125, 1.000)	1.000 (1.000, 1.000)

Abbreviations: AUC, area under curve; NPV, negative predictive value; PPV, positive predictive value; TIS, tumor in situ.

These 14 metabolites were confirmed using standard references.

^†^Age, sex, BMI, SBP, smoking, and alcohol drinking.

The presence of 56 metabolites would be challenging for ESCC screening. Therefore, we performed a stepwise logistic regression analysis in the validation dataset to determine the best subset of potential biomarkers among 56 metabolites (Figure 2D). We finally chose the subset of the top eight potential biomarkers, which has the highest AUC to predict ESCC screening‐positive subjects. Figure 2E–G indicates the combination of eight potential biomarkers has better screening and calibration performance than traditional risk factors. Tables S4 and S5 summarize the RF model combining eight potential metabolic biomarkers to discriminate ESCC screening‐positive subjects in the validation dataset. The combination of these eight potential biomarkers had AUC value of 0.945 (95% CI 0.895–0.978), with sensitivity of 89.1% (95% CI: 78.2–98.2%), specificity of 91.0% (95% CI: 83.3–98.7%), PPV of 88.3% (95% CI: 79.7–98.0%), and NPV of 92.7% (95% CI: 85.9–98.5%). The AUCs were 0.819 (95% CI 0.688–0.926), 0.951 (95% CI 0.900–0.989), 0.866 (95% CI 0.787–0.933) in esophagitis, dysplasia, and TIS and invasive cancer, respectively.

Table S6 presents reclassification table of individuals of predicted risk using risk factors only versus combined potential metabolic biomarkers. The net reclassification index (NRI) of risk factors‐only versus risk factors plus 14 potential metabolic biomarkers was 0.30 (95% CI 0.14–0.46, *p* < 0.001), while integrated discrimination improvement (IDI) was 0.11 (95% CI 0.06–0.16, *p* < 0.001). Consistent with results from receiver operating characteristic (ROC) analysis, prediction model composed of metabolites and risk factors was superior to all other models (Figure 1F and G). The NRI of risk factors‐only versus risk factors plus eight potential biomarkers was 1.18 (95% CI 0.97–1.37, *p* < 0.001), while IDI was 0.46 (95% CI 0.38–0.54, *p* < 0.001).

We used a secondary validation set from Shandong Tumor Hospital to validate the performance of two combinations of potential biomarkers. Consistent with the above results, the AUCs were 0.986 (95% CI 0.963–0.999) and 0.949 (95% CI 0.893–0.992) for the combination of 14 potential biomarkers and eight potential biomarkers, respectively (Table S7 and Figure S1).

Finally, we mapped 22 potential biomarkers based on KEGG database and MetaboAnalyst (Figure 3A).^7^ Four metabolic pathways were associated with ESCC at the FDR threshold of 0.05 and the pathway impact value threshold of 0.001. Figure 3B–D revealed evident disorders in tyrosine metabolism, tryptophan metabolism, phenylalanine, tyrosine and tryptophan biosynthesis, and phenylalanine metabolism.

**FIGURE 3:**
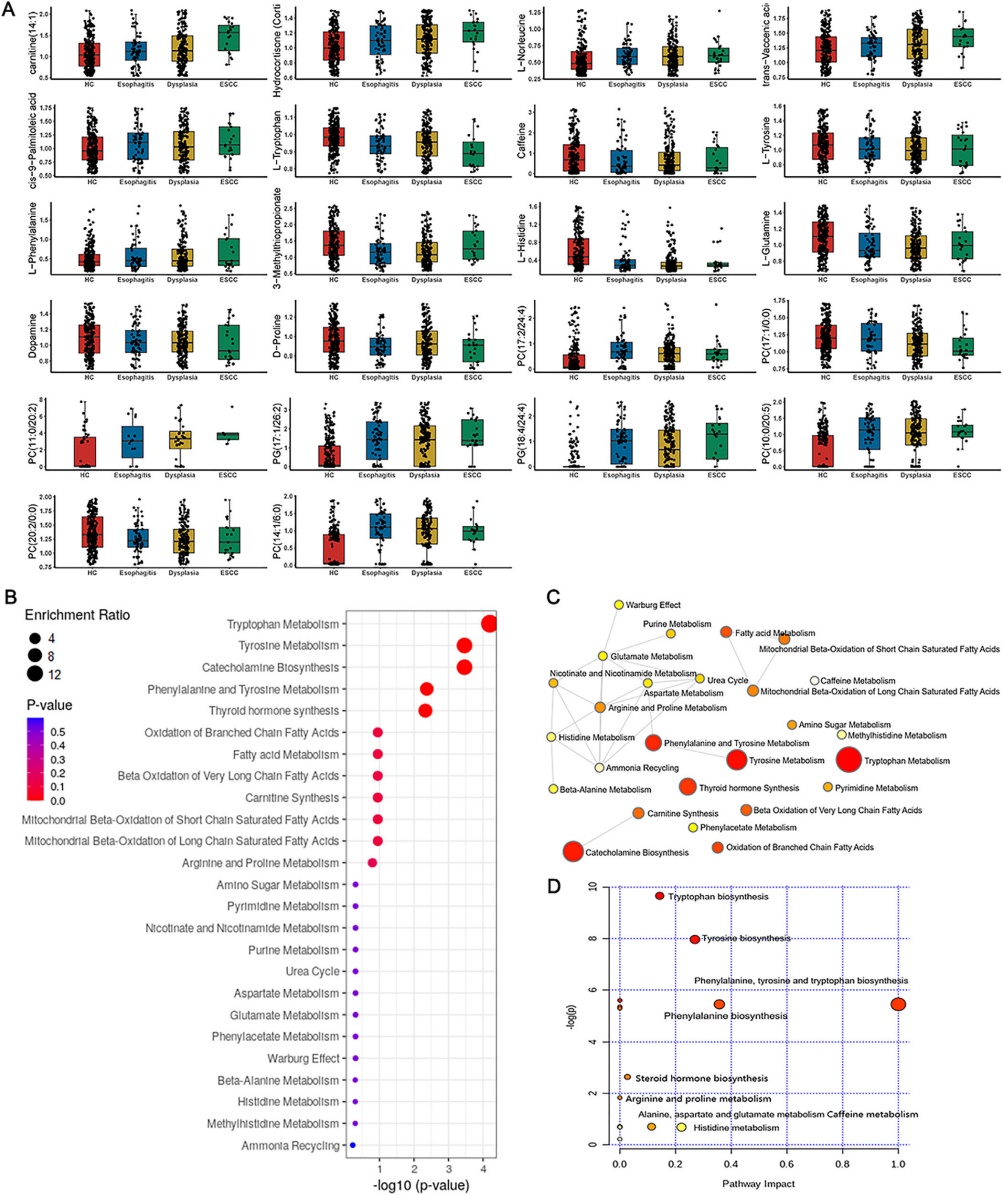
Enriched KEGG pathways analysis. (A) Boxplot of 22 differential metabolites; (B) metabolite sets enrichment overview; (C) network enrichment analysis; and (D) metabolic pathways associated with ESCC

In this untargeted metabolomics study, we conducted a population‐based screening study among adult subjects from high‐incidence area of China, with relatively large sample size. Currently, metabolomics studies for ESCC screening have been reported.^8^, ^9^, ^10^ However, these studies were restricted in small sample size and limited information on early ESCC screening. Based on this study, a new panel of differential metabolites were identified as potential biomarkers and showed good performance in ESCC screening.

Overall, we identified a new panel of differential metabolites as potential biomarkers to discriminate endoscopy screening‐positive population in this study. Risk reclassification was also improved significantly, compared with risk factors by the addition of metabolomic biomarkers. Better discrimination and calibration performance show these metabolites have utility in supporting clinical decisions and leads to the best decisions. A panel of serum metabolic biomarkers may be a valuable and invasive tool in ESCC early screening.

## FUNDING INFORMATION

National Natural Science Foundation of China, Grant Numbers: 81973147, 81573246, and 81673271; Cheeloo Young Scholars Program of Shandong University, Shandong University multidisciplinary Research and Innovation Team of Young Scholars, Grant Numbers: 2020QNQT11 and IFYT18034.

## CONFLICT OF INTEREST

The authors declare no conflict of interest.

## ETHICS APPROVAL STATEMENT

Study protocols were approved by the Ethics Committee of the Shandong Tumor Hospital, and written informed consent was obtained from all participants involved in this study (Ethics Approval ID: SDTHEC201305002).

## Supporting information

Supporting information
