# Supplementary material for: A serum metabolomics analysis reveals a panel of screening metabolic biomarkers for esophageal squamous cell carcinoma
Source: Clin Transl Med. 2021 May 6;11(5):e419. doi: 10.1002/ctm2.419 (PMC8101533; doi:10.1002/ctm2.419)
Supplement: Supplementary file 1 — Supporting information [file CTM2-11-e419-s001.docx]

**Supplementary Materials**

**A serum metabolomics analysis reveals a panel of screening metabolic biomarkers for esophageal squamous cell carcinoma**

Jiali Lv^1,2^†, Jialin Wang^3^†, Xiaotao Shen^4^, Jia Liu^5^, Deli Zhao^6^, Mengke Wei^1,2^, Xia Li^1,2^, Bingbing Fan^1,2^, Yawen Sun^3^, Fuzhong Xue^1,2^, Zheng-jiang Zhu^4#^, Tao Zhang^1,2#^

1. Department of Biostatistics, School of Public Health, Cheeloo College of Medicine, Shandong University, Jinan, Shandong, 250012, China

2. Institute for Medical Dataology, Cheeloo College of Medicine, Shandong University, Jinan, 250012, China

3. The Shandong Cancer Hospital Affiliated to Shandong University, Jinan 250117, China

4. Interdisciplinary Research Center on Biology and Chemistry, and Shanghai Institute of Organic Chemistry, Chinese Academy of Sciences, Shanghai 200032, China

5. Yanjing Medical College, Capital Medical University, Beijing 101300, China

6. Tumor Preventative and Therapeutic Base of Shandong Province, Feicheng People’s Hospital, Feicheng 271600, China

**Short Title:** serum metabolomics in ESCC screening

† These authors made equally contributions.

**# Correspondence & Reprints:**

Tao Zhang, MD, PhD

Department of Biostatistics, School of Public Health, Cheeloo College of Medicine, Shandong University, Jinan, Shandong, 250012, China

PO Box 100, 44 Wenhua Xi Rd, Jinan 250012, China.

Email: taozhang@sdu.edu.cn

Zheng-Jiang Zhu, PhD

Interdisciplinary Research Center on Biology and Chemistry, and Shanghai Institute of Organic Chemistry, Chinese Academy of Sciences, Shanghai 200032, China

Email: jiangzhu@sioc.ac.cn

**Study participants**

A total of 1104 participants were recruited at the Esophageal Cancer Screening Base of Shandong Province (City of Feicheng, Shandong, China) between June, 2013 and September, 2014. Study protocols were approved by the Ethics Committee of the Shandong Tumor Hospital and written informed consent was obtained from all participants involved in this study. Subjects aged 40~69 years voluntarily joined screening for esophageal cancer using endoscopy with mucosal iodine staining. The participants were randomly separated into training set (n=662) and validation set (n=442). In this study, the participants with normal esophageal mucosa (iodine-positive) were regarded as the healthy controls. Meanwhile, biopsies of the iodine-negative participants (ESCC screening-positive subjects, PRCS) were taken from the non-staining area of the mucosa, which were then underwent pathological evaluation to confirm and stage by two pathologists. Most ESCC screening-positive recruited at this Esophageal Cancer Screening Base were diagnosed with precursor lesions to ESCC, and were commonly considered to be high-risk population for ESCC. We also included 50 ESCC patients as secondary validation set to further validate the performance of candidate biomarkers.

**Data collection**

All participants completed a structured questionnaire designed to collect information previously found or suspected to be associated with ESCC. Body mass index (BMI) was calculated as self-reported weight in kilograms divided by self-reported height in meters squared. Smoking and alcohol drinking were defined as ever smoking cigarettes or ever drank alcohol, respectively.

**Serum Collection**

All participants were in an overnight fasting state and 5 mL of peripheral venous blood was taken in the morning. The blood was then allowed to clot for 30 minutes at 37°C water batch and followed by centrifugation at 3,000 rpm for 15 minutes. Then the serum supernatant was taken, immediately froze in liquid nitrogen, and stored at −80 °C until further analyses.

**Reagents and materials**

Ammonium fluoride (NH4F) was purchased from Fisher SIGMA (St. Louis, USA). LC-MS grade methanol (MeOH), water (H2O), acetonitrile (ACN), 0.1% formic acid (FA) in water and 0.1% FA in ACN were purchased from Honeywell (Muskegon, MI, USA).

**Sample preparation**

Serum samples were thawed at 4 °C on ice. Then 50 μL of serum sample was taken and placed in a 96-well plate, and every serum sample was extracted with 150 μL MeOH by using Bravo (Agilent, USA), then vortexed for 30 s and incubated for 2 hours at -20 °C to precipitate proteins. Then the 96 well plates were centrifuged for 20 min at 4,000 rpm and 4 °C. The resulting supernatant were transferred to LC MS vials (Agilent, USA) and stored at -80 °C prior to UPLC-QTOF/MS analysis.

**Quality control (QC) samples**

In this study, a total of 130 QC samples were used in both electrospray positive and negative modes. QC samples were created by mixing equal amounts of serum samples from five patients and five controls. The QC samples were run four times in randomized order within every analytical batch to monitor the stability of the analysis throughout the whole experimental procedures.

**UPLC-TOF-MS Analysis**

The UPLC-QTOF/MS analyses were performed using a UHPLC system (1290 series, Agilent Technologies, USA) coupled to a quadruple time-of-flight (QTOF) mass spectrometer (Agilent 6550 iFunnel Q-TOF, Agilent Technologies, USA). Waters ACQUITY UPLC HSS T3 columns (particle size, 1.8 μm; 100 mm (length) × 2.1 mm (i.d.)) were used for the LC separation and the column temperature was kept as 25 °C. The mobile phases A was 0.1% FA in water in positive mode (ESI+) or 0.5 mM NH4F in water in negative mode (ESI-), and B was 0.1% FA in ACN in positive mode or 100% ACN in negative mode. The linear gradient was set as follows: 0-1 min: 1% B, 1-8 min: 1% B to 100% B, 8-10 min: 100% B, 10-10.1 min: 100% B to 1% B, 10.1-12 min: 1% B. The acquisition rate was set as 4 spectra/s and the TOF mass range was set as m/z 50-1200 Da. The parameters of MS data acquisition were set as follows: sheath gas temperature, 400 °C; dry gas temperature, 250 °C; sheath gas flow, 12 L/min; dry gas flow, 16 L/min; capillary voltage, 3000 V in positive mode or -3000 V in negative mode, respectively; nozzle voltage, 0 V; and nebulizer pressure, 20 psi in positive or 40 psi in negative mode, respectively.

Tandem mass spectrometry (MS/MS) data acquisition was performed using another quadruple time-of-flight mass spectrometer (Triple TOF 5600+, AB SCIEX, USA). QC samples were used for MS/MS data acquisition. To expand the coverage of MS/MS spectra, the mass range were divided into 4 segments: 50-300 Da, 290-600 Da, 590-900 Da, 890-1200 Da. The acquired MS/MS spectra were matched against in-house tandem MS spectral library for metabolite identification. The source parameters were set as follows: GAS1, 60; GAS2: 60; CUR: 30; TEM: 600 °C; ISVF: 5500 V and -4500 V in positive and negative modes, respectively.

**Identification of Potential Biomarkers**

In the MS/MS experiments, a QTOF mass analyzer (6520, Agilent Technologies) was used to produce fragmentation patterns and structure information for biomarker candidates. Identification of each biomarker was based on mass data, retention time, MS/MS product ion patterns, and an online database query. The MS/MS fragmentation patterns of the biomarkers were compared to the spectral data of metabolites that had the same *m/z*, which were obtained from databases (HMDB (www.hmdb.ca) and METLIN (http://metlin.scripps.edu/). If available, confirmation with standards was performed by comparison of retention time, isotopic distribution, and fragments of reference standards (Sigma-Aldrich) with those obtained in real samples. The mass tolerance between the measured *m/z* values and the exact mass of the components of interest was set to 30 ppm.

**Data preprocessing and annotation**

Agilent raw data (.d) files were converted to the mzXML cross-platform open file format by using the freely available MSconvert software (ProteoWizard, <http://proteowizard.sourceforge.net>). All normalization process and further data analysis were performed using the R platform (version 3.6.0).

The raw UPLC-QTOF/MS ESI+ data were transformed to mzdata files by Mass Hunter Qualitative Analysis Software (Agilent Technologies) and then these files were imported to the *xcms* package in R for preprocessing. All the parameters were set as the default values. The preprocessing results generated a data matrix that consisted of the retention time, mass-to-charge ratio (*m/z*) values, and peak intensity. CAMERA in R was used for annotation of isotope peaks, adducts and fragments in the peak lists.

Only monoisotopic peaks annotated by CAMERA were selected for the subsequent statistical analyses. Peaks for which RSD >30% (337 out of 8182 in training set and 889 out of 8182 in validation set), were excluded from the analysis. Unnamed metabolites (7442 out of 7845 in the training set and 6880 out of 7283 in the validation set) were excluded from the analysis. Finally, 403 metabolic peaks were selected for subsequent analyses.

**Statistical analysis**

Characteristics across different groups were assessed using Student’s t test or Wilcoxon rank sum test as appropriate for continuous variables and $\chi^{2}$ test for categorical variables. Principal component analysis (PCA) was first used to reduce the dimensionality of the multidimensional dataset, and give a comprehensive view of the clustering trend for the multidimensional data.

Partial least-squares discriminant analysis (PLS-DA) was used to understand global metabolic changes between healthy controls and ESCC screening positive population, and variable importance in the projection (VIP values) were calculated in PLS-DA model as well. Validation plot was used to assess the validity of PLS-DA model by comparing the goodness of fit (R2 and Q2) of the PLS-DA models with the goodness of fit of 200 Y-permutated models. Meanwhile, the nonparametric Kruskal-Wallis rank sum test was performed to determine the significance of each metabolite, and the relevant false discovery rates (FDR) based on the p-values were estimated. Potential metabolic biomarker was selected when the value of its VIP was more than 1 and FDR was less than 0.05. Random forest (R package ‘‘*randomforest*”) was used to build prediction model.

To evaluate the classification performance, the area under the receiver operating characteristic curve (AUC) was computed. Net reclassification index (NRI), integrated discrimination improvement (IDI) were also calculated to explore the predictive value of potential biomarkers. Then, we performed decision curve analysis (DCA analysis) for further evaluating whether alternative metabolic screening strategies have advantages over risk factors. All statistical analyses were performed on the R platform (version 3.6.0).

**Supplement Table S1.** Differential metabolites identified using standard references

| Metabolites^†^ | Fold change^‡^ | Direction | *P*-value^‖^ | FDR | VIP |
| --- | --- | --- | --- | --- | --- |
| D-Proline | 0.892 | Down | <0.001 | <0.001 | 1.074 |
| Dopamine | 0.879 | Down | <0.001 | 0.001 | 1.124 |
| L-Glutamine | 0.820 | Down | <0.001 | <0.001 | 1.916 |
| L-Histidine | 0.379 | Down | <0.001 | <0.001 | 2.246 |
| 3-Methylthiopropionate | 0.637 | Down | <0.001 | <0.001 | 1.867 |
| L-Phenylalanine | 2.674 | Up | <0.001 | <0.001 | 1.141 |
| L-Tyrosine | 0.801 | Down | <0.001 | <0.001 | 1.574 |
| Caffeine | 0.454 | Down | <0.001 | <0.001 | 1.472 |
| L-Tryptophan | 0.944 | Down | 0.001 | 0.003 | 1.276 |
| cis-9-Palmitoleic acid | 1.256 | Up | <0.001 | <0.001 | 1.465 |
| trans-Vaccenic acid | 1.163 | Up | <0.001 | <0.001 | 1.468 |
| L-Norleucine | 0.725 | Down | 0.281 | 0.412 | 1.086 |
| Hydrocortisone (Cortisol) | 1.135 | Up | <0.001 | <0.001 | 1.564 |
| Carnitine (14:1) | 1.157 | Up | 0.036 | 0.096 | 1.126 |

FDR means p value adjusted using False Discovery Rate, VIP means variable importance in projection.

‡ Fold change means the ratio of the mean value of ESCC screening-positive subjects relative to healthy controls.

‖ Adjusted for age, sex, BMI, SBP, smoking and alcohol drinking.

**Supplement Table S2.** ROC analysis of random forest model combing 14 metabolites to predict ESCC screening-positive subjects

|  | N | PPV% ^1^ | NPV% ^1^ | PPV% ^2^ | NPV% ^2^ |
| --- | --- | --- | --- | --- | --- |
| Risk factors^†^ |  | 0.02 | 100.00 | 0.17 | 99.95 |
| Metabolites |  | 0.03 | 100.00 | 0.29 | 99.98 |
| Metabolites & Risk factors^†^ |  | 0.04 | 100.00 | 0.35 | 99.97 |
| Metabolites (stages) |  |  |  |  |  |
| Esophagitis | 56 | 0.02 | 100.00 | 0.24 | 99.97 |
| Dysplasia | 106 | 0.03 | 100.00 | 0.30 | 99.98 |
| TIS & Invasive cancer | 8 | 0.10 | 100.00 | 0.99 | 100.00 |

^1^ Assume ESCC prevalence as 1/1000 in city of Feicheng.

^2^ Assume ESCC prevalence as 1/100 in high-risk population or in clinical practice.

† Age, sex, BMI, SBP, smoking and alcohol drinking.

PPV = positive predictive value, NPV = negative predictive value, TIS = tumor in situ.

These 14 metabolites were confirmed using standard references.

**Supplement Table S3.** Differential metabolites interpreted according to their MS/MS spectra

| Metabolites | Fold change^‡^ | Direction^§^ | *P*-value^‖^ | FDR | VIP |
| --- | --- | --- | --- | --- | --- |
| PC (17:1/0:0) | 0.848 | Down | < 0.001 | < 0.001 | 1.652 |
| PC (20:2/0:0) | 0.845 | Down | < 0.001 | < 0.001 | 1.486 |
| PC (14:1/6:0) | 0.710 | Down | 0.967 | 0.975 | 1.524 |
| PC (10:0/20:5) | 0.179 | Down | 0.154 | 0.273 | 1.508 |
| PC (11:0/20:2) | 0.799 | Down | 0.626 | 0.733 | 1.462 |
| PC (17:2/24:4) | 0.475 | Down | 0.400 | 0.526 | 1.552 |
| PG (18:4/24:4) | 4.994 | Up | < 0.001 | < 0.001 | 2.759 |
| PG (17:1/26:2) | 0.823 | Down | 0.538 | 0.654 | 1.504 |

FDR = FDR adjusted p-value, VIP = The variable importance in projection.

‡ Fold change was calculated from the ratio of the mean values of ESCC screening-positive subjects relative to healthy controls.

‖ Adjusted for age, sex, BMI, SBP, smoking and alcohol drinking.

§ “Down” means a relative low concentration compared to the healthy controls, while “Up” means a relative high concentration compared to the healthy controls.

**Supplement Table S4.** ROC analysis of random forest model combing 8 metabolites to predict ESCC screening-positive subjects in the validation data

|  | N | AUC | Sensitivity | Specificity | PPV | NPV |
| --- | --- | --- | --- | --- | --- | --- |
| Risk factors^†^ |  | 0.643 (0.541, 0.734) | 0.756 (0.533, 0.933) | 0.557 (0.341, 0.761) | 0.466 (0.396, 0.575) | 0.817 (0.738, 0.931) |
| Metabolites |  | 0.945 (0.895, 0.978) | 0.891 (0.782, 0.982) | 0.910 (0.833, 0.987) | 0.883 (0.797, 0.980) | 0.927 (0.859, 0.985) |
| Metabolites & Risk factors^†^ |  | 0.946 (0.895, 0.985) | 0.927 (0.836, 0.982) | 0.910 (0.821, 0.974) | 0.879 (0.790, 0.962) | 0.947 (0.892, 0.987) |
| Metabolites (stages) |  |  |  |  |  |  |
| Esophagitis | 56 | 0.819 (0.688, 0.926) | 0.750 (0.550, 0.900) | 0.924 (0.835, 0.987) | 0.700 (0.520, 0.909) | 0.935 (0.889, 0.975) |
| Dysplasia | 106 | 0.951 (0.900, 0.989) | 0.968 (0.903, 1.000) | 0.928 (0.867, 0.976) | 0.833 (0.732, 0.939) | 0.987 (0.961, 1.000) |
| TIS & Invasive cancer | 8 | 0.866 (0.787, 0.933) | 1.000 (1.000, 1.000) | 0.866 (0.780, 0.927) | 0.154 (0.100, 0.250) | 1.000 (1.000, 1.000) |

AUC = area under curve, PPV = positive predictive value, NPV = negative predictive value, TIS = tumor in situ.

These 8 metabolites were interpreted according to their MS/MS spectra.

† Age, sex, BMI, SBP, smoking and alcohol drinking.

**Supplement Table S5.** ROC analysis of random forest model combing 8 metabolites to predict ESCC screening-positive subjects

|  | N | PPV% ^1^ | NPV% ^1^ | PPV% ^2^ | NPV% ^2^ |
| --- | --- | --- | --- | --- | --- |
| Risk factors^†^ |  | 0.02 | 100.00 | 0.17 | 99.96 |
| Metabolites |  | 0.10 | 100.00 | 0.98 | 99.99 |
| Metabolites & Risk factors^†^ |  | 0.10 | 100.00 | 1.01 | 99.99 |
| Metabolites (stages) |  |  |  |  |  |
| Esophagitis | 56 | 0.09 | 100.00 | 0.93 | 99.98 |
| Dysplasia | 106 | 0.12 | 100.00 | 1.19 | 100.00 |
| TIS & Invasive cancer | 8 | 0.71 | 100.00 | 0.71 | 100.00 |

^1^ Assume ESCC prevalence as 1/1000 in city of Feicheng.

^2^ Assume ESCC prevalence as 1/100 in high-risk population or in clinical practice.

† Age, sex, BMI, SBP, smoking and alcohol drinking.

PPV = positive predictive value, NPV = negative predictive value, TIS = tumor in situ.

These 8 metabolites were interpreted according to their MS/MS spectra.

**Supplement Table S6.** Reclassification table of individuals of predicted risk using risk factors only versus combined potential biomarkers

|  | **Risk factors and 14 metabolites** | | | |  | **Risk factors and 8 metabolites** | | | |
| --- | --- | --- | --- | --- | --- | --- | --- | --- | --- |
| **Risk factors only** | **< 25%** | **25%~75%** | **> 75%** | **Reclassified%** |  | **< 25%** | **25%~75%** | **> 75%** | **Reclassified%** |
| **< 25%** |  |  |  |  |  |  |  |  |  |
| Total | 13 | 1 | 0 | 7 |  | 8 | 5 | 1 | 43 |
| Cases | 4 | 1 | 0 | 20 |  | 2 | 2 | 1 | 60 |
| Controls | 9 | 0 | 0 | 0 |  | 6 | 3 | 0 | 33 |
| **25%~75%** |  |  |  |  |  |  |  |  |  |
| Total | 29 | 83 | 7 | 30 |  | 60 | 29 | 30 | 76 |
| Cases | 4 | 42 | 4 | 16 |  | 2 | 21 | 27 | 58 |
| Controls | 25 | 41 | 3 | 41 |  | 58 | 8 | 3 | 88 |
| **> 75%** |  |  |  |  |  |  |  |  |  |
| Total | 0 | 0 | 0 | - |  | 0 | 0 | 0 | - |
| Cases | 0 | 0 | 0 | - |  | 0 | 0 | 0 | - |
| Controls | 0 | 0 | 0 | - |  | 0 | 0 | 0 | - |
| **NRI (95% CI)** | 0.30 (95%CI 0.14~0.46); p <0.001 | | | |  | 1.18 (95%CI 0.97~1.37); p <0.001 | | | |
| **IDI (95% CI)** | 0.11 (95%CI 0.06~0.16); p <0.001 | | | |  | 0.46 (95%CI 0.38~0.54); p <0.001 | | | |

NRI = net reclassification index; IDI = integrated discrimination improvement.

Low risk <25%, intermediate risk 25–75%, high risk >75%.

**Supplement Table S7.** ROC analysis of random forest models to predict ESCC screening-positive subjects in secondary validation set

|  | N | AUC | Sensitivity | Specificity | PPV | NPV |
| --- | --- | --- | --- | --- | --- | --- |
| **14 Markers** |  |  |  |  |  |  |
| ESCC | 50 | 0.986 (0.963, 0.999) | 1.000 (0.938, 1.000) | 0.926 (0.864, 1.000) | 0.727 (0.593, 1.000) | 1.000 (0.988, 1.000) |
| Stage TIS–Ⅱ | 21 | 0.981 (0.942, 1.000) | 1.000 (1.000, 1.000) | 0.926 (0.840, 1.000) | 0.538 (0.350, 1.000) | 1.000 (1.000, 1.000) |
| Stage Ⅲ | 29 | 0.997 (0.989, 1.000) | 1.000 (1.000, 1.000) | 0.987 (0.935, 1.000) | 0.933 (0.737, 1.000) | 1.000 (1.000, 1.000) |
| **8 Markers** |  |  |  |  |  |  |
| ESCC | 50 | 0.949 (0.893, 0.992) | 0.875 (0.750, 1.000) | 0.963 (0.691, 1.000) | 0.824 (0.390, 1.000) | 0.976 (0.950, 1.000) |
| Stage TIS–Ⅱ | 21 | 0.990 (0.968, 1.000) | 1.000 (1.000, 1.000) | 0.963 (0.889, 1.000) | 0.700 (0.438, 1.000) | 1.000 (1.000, 1.000) |
| Stage Ⅲ | 29 | 0.935 (0.867, 0.984) | 0.929 (0.714, 1.000) | 0.857 (0.662, 1.000) | 0.545 (0.350, 1.000) | 0.985 (0.949, 1.000) |

AUC = area under curve, PPV = positive predictive value, NPV = negative predictive value, TIS = tumor in situ.

**Supplement Table S8.** KEGG pathway enrichment analysis

| Pathway | TC | Hits | *P*-value | FDR | Impact |
| --- | --- | --- | --- | --- | --- |
| Tryptophan metabolism | 41 | 1 | < 0.001 | 0.001 | 0.143 |
| Tyrosine metabolism | 42 | 2 | < 0.001 | 0.003 | 0.269 |
| Cysteine and methionine metabolism | 33 | 1 | 0.004 | 0.014 | < 0.001 |
| Phenylalanine, tyrosine and tryptophan biosynthesis | 4 | 2 | 0.004 | 0.014 | 1.000 |
| Phenylalanine metabolism | 10 | 2 | 0.004 | 0.014 | 0.357 |
| Ubiquinone and other terpenoid-quinone biosynthesis | 9 | 1 | 0.005 | 0.014 | < 0.001 |
| Aminoacyl-tRNA biosynthesis | 48 | 5 | 0.005 | 0.014 | < 0.001 |
| Steroid hormone biosynthesis | 85 | 1 | 0.072 | 0.170 | 0.027 |
| Arginine and proline metabolism | 38 | 1 | 0.160 | 0.338 | < 0.001 |
| Alanine, aspartate and glutamate metabolism | 28 | 1 | 0.494 | 0.531 | 0.113 |
| Arginine biosynthesis | 14 | 1 | 0.494 | 0.531 | < 0.001 |
| Purine metabolism | 65 | 1 | 0.494 | 0.531 | < 0.001 |
| Pyrimidine metabolism | 39 | 1 | 0.494 | 0.531 | < 0.001 |
| D-Glutamine and D-glutamate metabolism | 6 | 1 | 0.494 | 0.531 | < 0.001 |
| Glyoxylate and dicarboxylate metabolism | 32 | 1 | 0.494 | 0.531 | < 0.001 |
| Nitrogen metabolism | 6 | 1 | 0.494 | 0.531 | < 0.001 |
| Histidine metabolism | 16 | 1 | 0.503 | 0.531 | 0.221 |
| beta-Alanine metabolism | 21 | 1 | 0.503 | 0.531 | < 0.001 |
| Caffeine metabolism | 10 | 1 | 0.801 | 0.801 | < 0.001 |

TC is the total number of compounds in the pathway.

Hits is the actually matched number from our data.

FDR is the p value adjusted using False Discovery Rate.

Impact is the pathway impact value calculated from pathway topology analysis.

**Supplement Table S9.** Detailed information about random forest models composed 14 metabolic biomarkers to predict ESCC screening-positive subjects

| Model | ntree | mtry | Error rate | Class error (HC group) | Class error (PRCS group) |
| --- | --- | --- | --- | --- | --- |
| Risk factors^†^ | 1000 | 2 | 33.01% | 0.184 | 0.544 |
| Metabolites | 1000 | 3 | 30.81% | 0.222 | 0.420 |
| Metabolites & Risk factors^†^ | 1000 | 4 | 29.13% | 0.119 | 0.583 |
| Metabolites (by stages) | 1000 | 3 | 15.72% | 0.000 | 1.000 |
| Esophagitis | 1000 | 3 | 24.24% | 0.074 | 0.667 |
| Dysplasia | 1000 | 3 | 3.06% | 0.000 | 1.000 |
| TIS & Invasive cancer | 1000 | 2 | 33.01% | 0.184 | 0.544 |

ntree means number of trees grown, mtry means number of predictors sampled for splitting at each node, Error rate means error rates of the prediction on the input data by out-of-bag estimate, Class error (HC group) means error rates in HC group by the confusion matrix of the prediction (based on OOB data), Class error (PRCS group) means error rates in PRCS group by the confusion matrix of the prediction (based on OOB data).


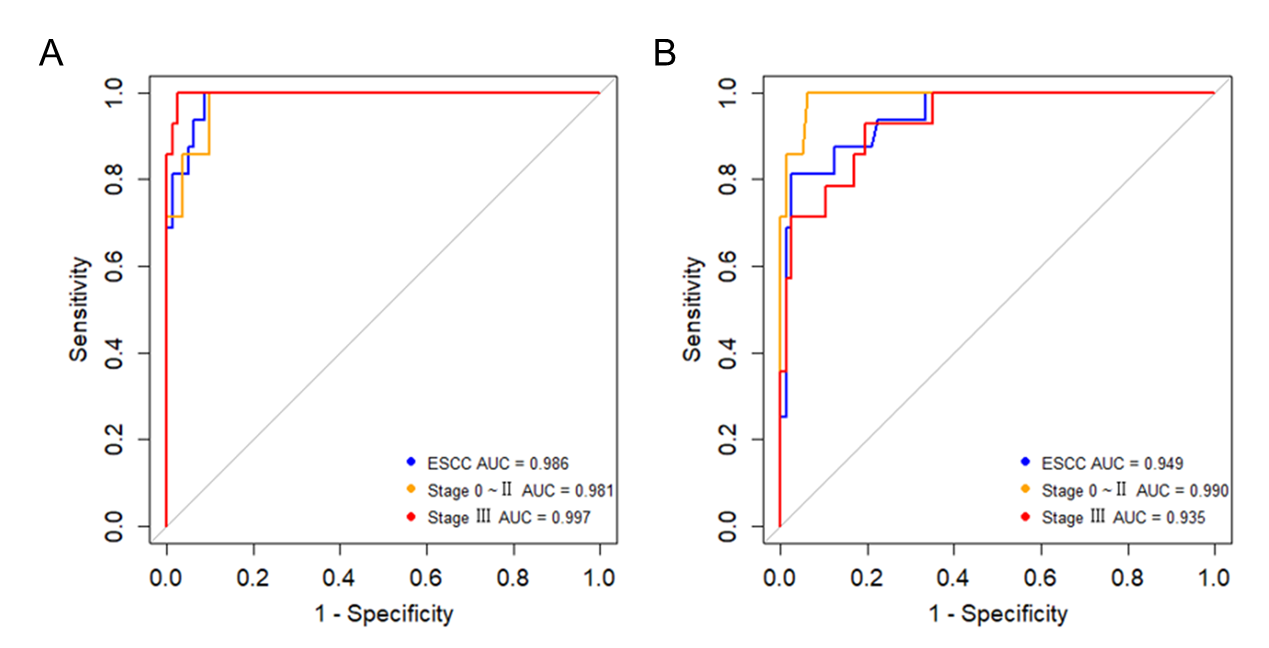


**Supplement Figure S1.** ROC analysis of random forest model composed biomarkers to predict ESCC screening-positive subjects in secondary validation set. (A) for metabolites were confirmed using standard references; (B) for metabolites were interpreted according to their MS/MS spectra.
